# Targeted treatment of chondrosarcoma with a bacteriophage-based particle delivering a secreted tumor necrosis factor-related apoptosis-inducing ligand

**DOI:** 10.1016/j.omton.2024.200805

**Published:** 2024-04-24

**Authors:** Aitthiphon Chongchai, Kaoutar Bentayebi, Grace Chu, Wenqing Yan, Sajee Waramit, Thanyaluck Phitak, Prachya Kongtawelert, Peraphan Pothacharoen, Keittisak Suwan, Amin Hajitou

**Affiliations:** 1 Cancer Phage Therapy Laboratory, Department of Brain Sciences, Imperial College London, W12 0NN London, UK; 2 Thailand Excellence Centre for Tissue Engineering and Stem Cells, Department of Biochemistry, Faculty of Medicine, Chiang Mai University, Chiang Mai 50200, Thailand; 3 Medical Biotechnology Laboratory, Rabat Medical and Pharmacy School, Mohammed V University, Rabat 10100, Morocco

**Keywords:** MT: Regular Issue, chondrosarcoma, bacteriophage, tumor targeting, targeted cytokine therapy, TRAIL, systemic delivery, gene therapy, cancer treatment, targeted gene delivery

## Abstract

Chondrosarcoma (CS) is a malignant cartilage-forming bone tumor that is inherently resistant to chemotherapy and radiotherapy, leaving surgery as the only treatment option. We have designed a tumor-targeted bacteriophage (phage)-derived particle (PDP), for targeted systemic delivery of cytokine-encoding transgenes to solid tumors. Phage has no intrinsic tropism for mammalian cells; therefore, it was engineered to display a double cyclic RGD4C ligand on the capsid to target tumors. To induce cancer cell death, we constructed a transgene cassette expressing a secreted form of the cytokine tumor necrosis factor-related apoptosis-inducing ligand (sTRAIL). We detected high expression of αvβ3 and αvβ5 integrin receptors of the RGD4C ligand, and of the TRAIL receptor-2 in human CS cells (SW1353), but not in primary normal chondrocytes. The RGD4C.PDP-*Luc* particle carrying a luciferase reporter gene, *Luc*, effectively and selectively mediated gene delivery to SW1353 cells, but not primary chondrocytes. Transduction of SW1353 cells with RGD4C.PDP*-sTRAIL* encoding a human sTRAIL, resulted in the expression of TRAIL and subsequent cell death without harming the normal chondrocytes. Intravenous administration of RGD4C.PDP-*sTRAIL* to mice with established human CS resulted in a decrease in tumor size and tumor viability. Altogether, RGD4C.PDP-*sTRAIL* can be used to target systemic treatment of CS with the sTRAIL.

## Graphical abstract

**Figure:**
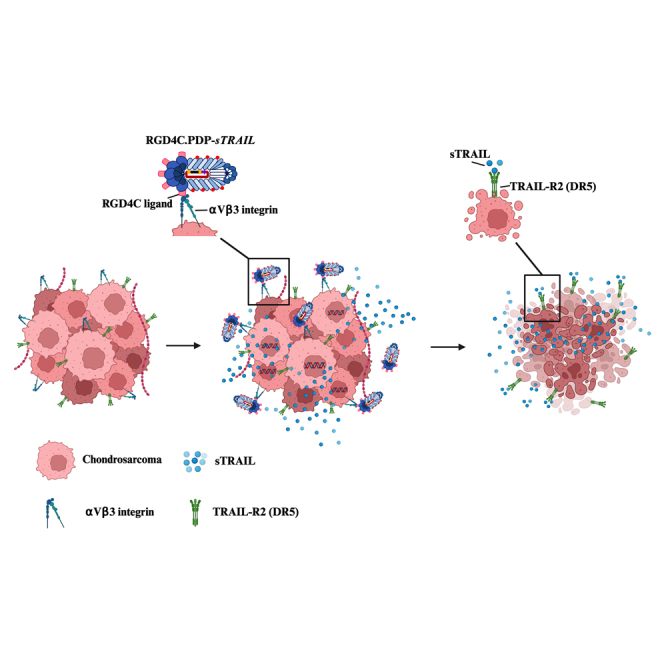

## Teaser

Hajitou, Suwan, and colleagues describe a novel treatment strategy against human CS using a phage-guided gene delivery particle encoding a secreted TRAIL cytokine into the tumor microenvironment. *In vitro*, targeted TRAIL delivery resulted in CS cell death. *In vivo*, intravenous treatment inhibited CS growth and tumor viability in CS-bearing mice.

## Introduction

Chondrosarcoma (CS) is a malignant cartilage-forming bone tumor that contributes 20%–27% of primary malignant bone neoplasms and mainly occurs in the long tubular bones. Patients with CS experience pain, swelling, and a palpable soft tissue mass.^1^ These tumors are resistant to chemotherapy and radiation due to their low proportion of dividing cells, slow division rate, poor vascularization, and the compact matrix densely composed of hyaline cartilaginous, thus, leaving surgical excision is the only current effective treatment option.^2^ Moreover, local recurrence and metastases might happen in certain patients. More than 90% of CS is conventional, and, of these, 5%–10% are grade 3 and have a significant risk for metastasis.^3^ The prognosis for patients with metastatic CS is frequently poor^4^ and results in death. The development of effective treatment approaches is an urgent need since the clinical management of CS is extremely challenging. Gene therapy is a promising treatment strategy against cancer and has been attempted for the last 30 years with more than 67% of clinical trials designed to treat cancer.^5^ Cancer gene therapy aims at delivering therapeutic genes to express anti-cancer proteins within the tumor microenvironment. There have been, however, a handful of gene therapy investigations of CS.^6^^,^^7^ Moreover, gene therapy has faced major challenges hindering clinical application, mainly the lack of an ideal gene delivery vector efficient via systemic clinical routes, such as intravenously. Local delivery can be used to show proof of efficacy, but real clinical benefit can only occur via systemic administration, mainly for the treatment of metastatic cancer, which is associated with 90% of cancer deaths. Repeated dosing has also been a problem because of the immunogenicity of vectors.^8^ To improve systemic gene therapy for cancer, we have used phages to design vectors for systemic gene delivery. Phages, prokaryotic viruses that infect bacteria only, have no tropism for mammalian tissues; subsequently, their systemic administration does not require the ablation of a mammalian tropism, unlike existing eukaryotic viral vectors.^9^^,^^10^ A key strategy of our vector model is the design of hybrid prokaryotic-eukaryotic viral vectors combining the attributes of these two virus kingdoms. In 2006, we reported a chimeric systemic intravenous gene delivery system to target tumors selectively by using the harmless and non-pathogenic filamentous M13 phage.^11^ In that vector, the M13 phage carries an adeno-associated virus (AAV2)-based transgene expression cassette inserted within the phage genome. Since the phage does not infect mammalian cells, we engineered its capsid to display the double cyclic CDCRGDCFC (RGD4C) ligand that binds the αvβ3 and αvβ5 integrins cell surface receptors. These two integrin heterodimers are overexpressed in tumors, both on tumor cells and tumor angiogenic and abnormal vasculature, but barely detectable in healthy tissues and normal bloods vessels.^11^^,^^12^^,^^13^^,^^14^ This vector safely targets various preclinical models of human cancer in rodents and natural cancers in dogs.^14^

Recently, we have refined this delivery technology by designing a second generation in which the phage genome was removed to produce a reduced size chimera between the phage capsid and the sole recombinant DNA of AAV2 or inverted terminal repeat (*ITR*)-flanked transgene cassettes. In this second-generation M13 phage vector, the M13 *f1* origin of replication is the only remaining phage *cis* element to allow replication of the AAV-based transgene cassettes in the bacteria host and its packaging by the tumor-targeting phage coat proteins displaying the RGD4C ligand. The phage coat proteins are provided via a helper phage infection of the host manufacturing bacteria.^15^^,^^16^ Expression of αvβ3 and αvβ5 integrins, receptors of RGD4C, on the cell surface of human CS has been reported.^17^

We previously used this second-generation vector to show application in targeted cancer immunotherapy by guided delivery of cytokine-encoding genes resulting in safe and efficient cytokine therapy of cancer.^15^ Moreover, we reported in a previous study that display the histidine-rich endosomal escape peptide (EEP), GLFHAIAHFIHGGWHGLIHGWYG (H5WYG) on the M13 phage capsid improved endosomal escape of phage vectors and subsequently enhanced gene delivery.^18^ Thus, herein, we have capitalized on our previous studies and constructed a phage-derived particle (PDP) based on the second-generation M13 vector for targeted systemic cytokine gene therapy for CS treatment. The PDP system displays five copies of the RGD4C ligand on the pIII minor coats proteins to allow selective tumor homing, and numerous copies of the H5WYG EEP on the recombinant pVIII major coat proteins for endosomal escape. Moreover, we generated a PDP particle carrying a redesigned DNA sequence of the cytokine tumor necrosis factor-related apoptosis-inducing ligand (TRAIL) encoding for a secreted TRAIL. TRAIL is a member of the tumor necrosis factor family of ligands. TRAIL causes apoptosis primarily in tumor cells, but not normal cells,^19^ by binding to its death receptors expressed on tumor cells, but absent on normal cells. TRAIL and its death receptors have been used to target selective tumor death in cancer patients, such as using monoclonal antibodies, mapatumumab, as agonistic to the TRAIL death receptor 1.^20^ However, the TRAIL cytokine protein has a very short half-life, and its systemic administration to achieve a clinical benefit, has been challenging.^21^ We postulated that targeted systemic delivery of TRAIL to CS by PDP carrying a TRAIL encoding DNA is solution. In this study, we report treatment of human CS cells with RGD4C.PDP-*sTRAIL* resulted in the selective production of TRAIL and subsequent tumor cell killing, with no effect on normal human primary chondrocytes. Targeted systemic cytokine gene therapy in tumor-bearing mice resulted in the destruction of human CS.

## Results

### Human CS cells express αvβ3 and αvβ5 integrin receptors for tumor targeting, but not normal human primary chondrocytes

**Figure 1:**
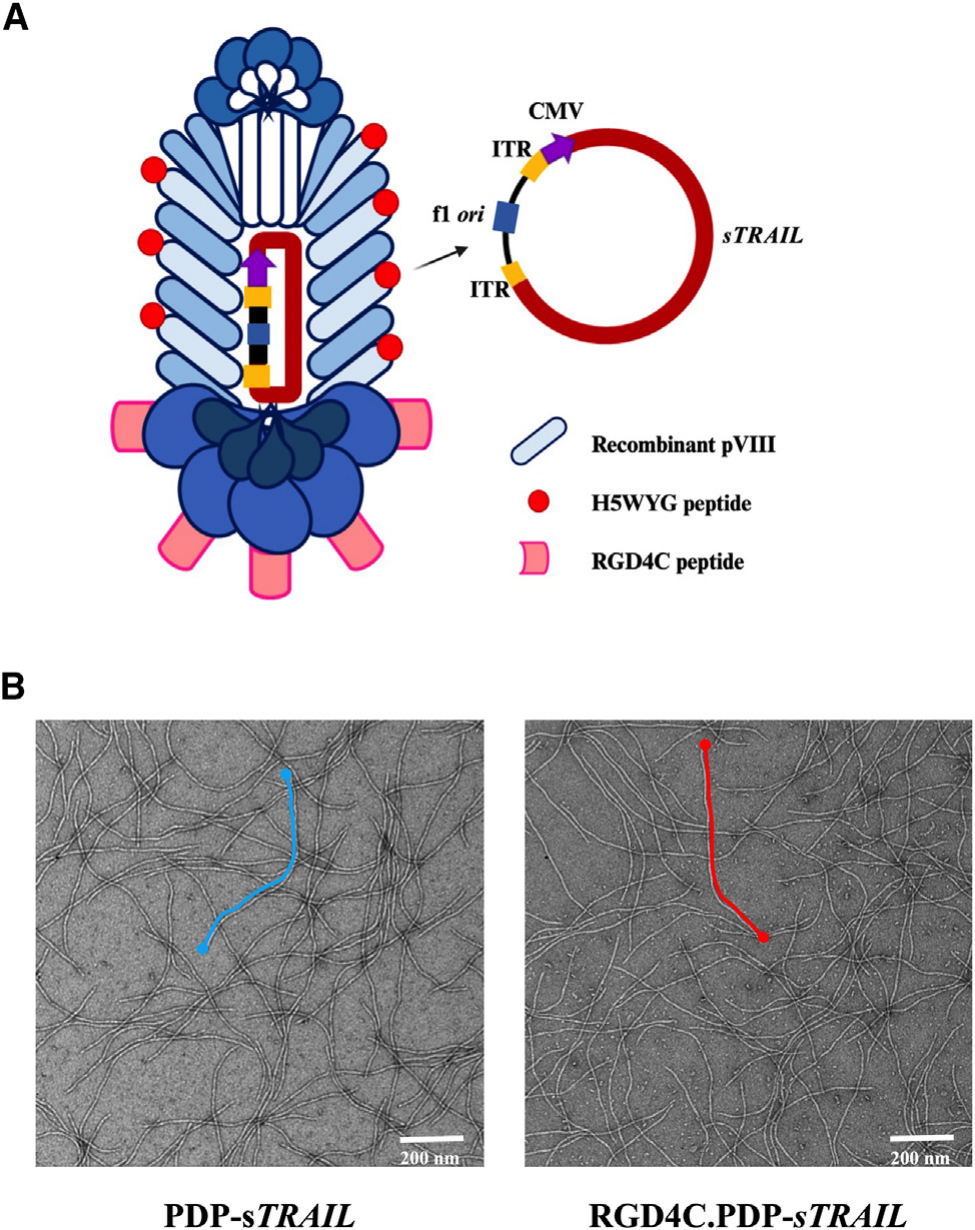
Structure of bacteriophage (phage)-derived particle (PDP) (A) Schematic representation of the RGD4C.PDP-*sTRAIL* particles. PDP comprises three alterations: (1) the pIII minor coat proteins display five copies of the double cyclic RGD4C ligand, (2) the recombinant pVIII major coat proteins express the EEP H5WYG, and (3) the PDP capsid packages a mammalian transgene cassette for s*TRAIL* expression, flanked by AAV2 ITRs. The *f1* ori (phage origin of replication), which enables single-stranded DNA replication and packaging into the phage capsid, is the only remaining phage genetic sequence. (B) Electron microscopic images of the PDP particles, targeted RGD4C.PDP-*sTRAIL* and non-targeted PDP-*sTRAIL* are shown. Scale bar, 200 nm.

**Figure 2:**
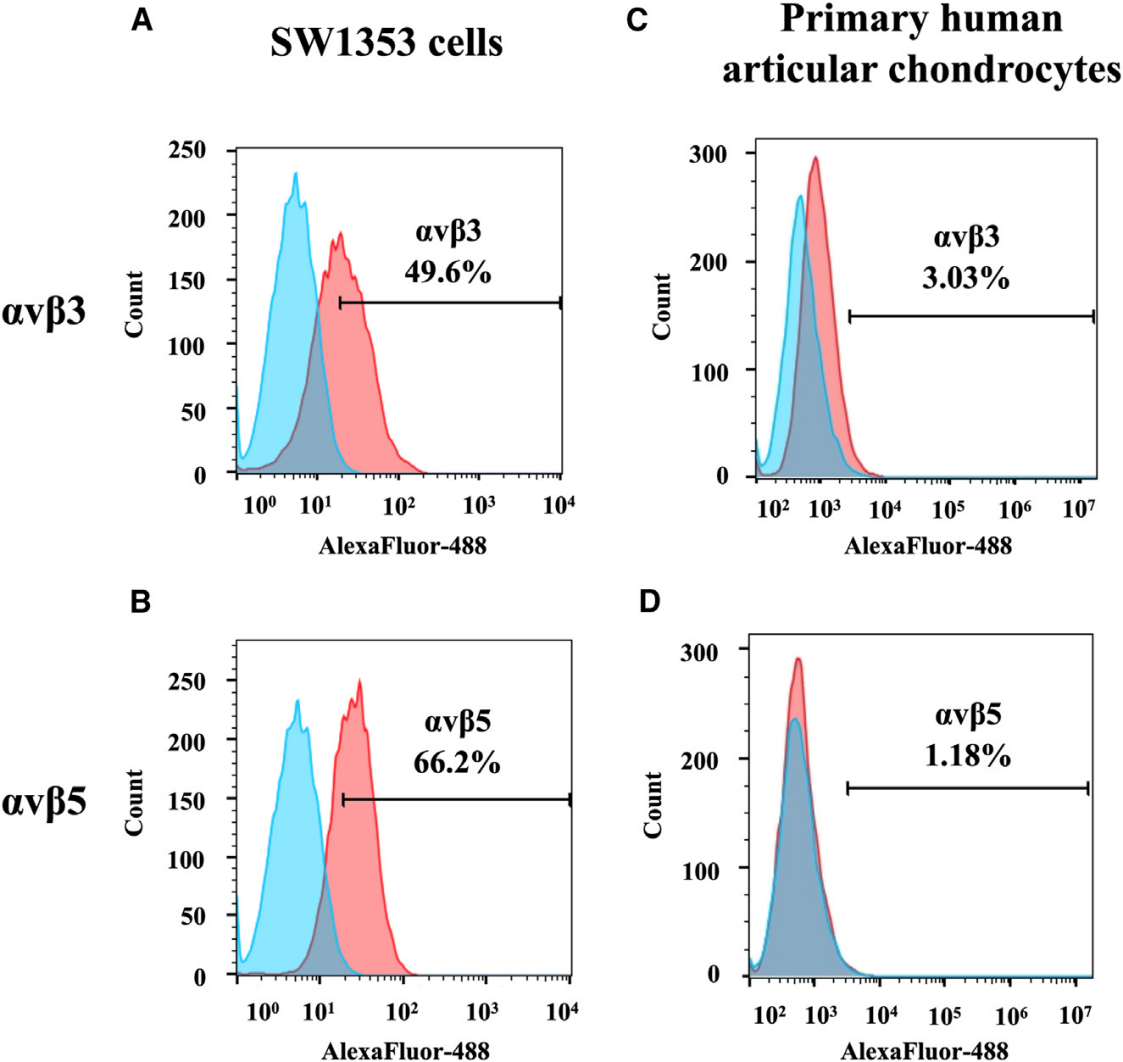
Analysis of expression of αvβ3 and αvβ5 integrin receptors on human CS cells (SW1353) and primary HACs (A and B) SW1353 cells were stained with antibodies against the heterodimer αvβ3 (A) or αvβ5 (B). The expression was analyzed by flow cytometry and FlowJo software. (C and D) HAC cells were also included for staining of αvβ3 (C) and αvβ5 (D) integrins. Cells incubated with the secondary antibody alone were used as controls. The FACS results show the difference between stained and control groups.

In this study, we aimed to use the PDP particle as a gene delivery platform for human CS. The PDP displays five copies of the double cyclic RGD4C ligand on the pIII minor coat proteins to serve as a tumor-targeting ligand. The RGD4C binds specifically to αvβ3 or αvβ5 integrin receptors on the surface of cancer cells.^12^^,^^22^^,^^23^ Moreover, RGD4C.PDP was modified to (1) display the EEP, H5WYG, on the recombinant pVIII coat proteins^18^ and to (2) carry an (AAV2 *ITR*)-flanked transgene cassette encoding a secreted sTRAIL^15^ (Figure 1A). Following vector production, PDP particles carrying the *sTRAIL* DNA sequence were visualized using transmission electron microscopy, as we have reported^15^ (Figure 1B). Next, we measured the length of the targeted RGD4C.PDP and non-targeted PDP, but did not find any significant differences between the two vectors (Figure S1). The human CS cell line (SW1353) and primary human articular chondrocytes (HACs) were stained with antibodies against the heterodimers αvβ3 and αvβ5 integrins and analyzed by flow cytometry. Expression of the αvβ3 and αvβ5 integrins on the cell surface of SW1353 was detected in approximately 50% and 66% of tumor cells, respectively (Figures 2A and 2B). Interestingly, primary HACs barely express αvβ3 and αvβ5 integrins, which were only observed in 3% and 1% of the cell population, respectively (Figures 2C and 2D). We further confirmed the expression of these integrins with immunofluorescent staining and found strong expression of the αv and β5 integrins on SW1353 cells (Figure S2), whereas no expression of these three integrin subtypes was detected on primary HACs (Figure S2). Therefore, SW1353 cells can be utilized as a CS model for PDP-guided delivery of the *sTRAIL* gene.

### PDP efficiently targets and delivers transgene expression to human CS cells, but not normal HACs

**Figure 3:**
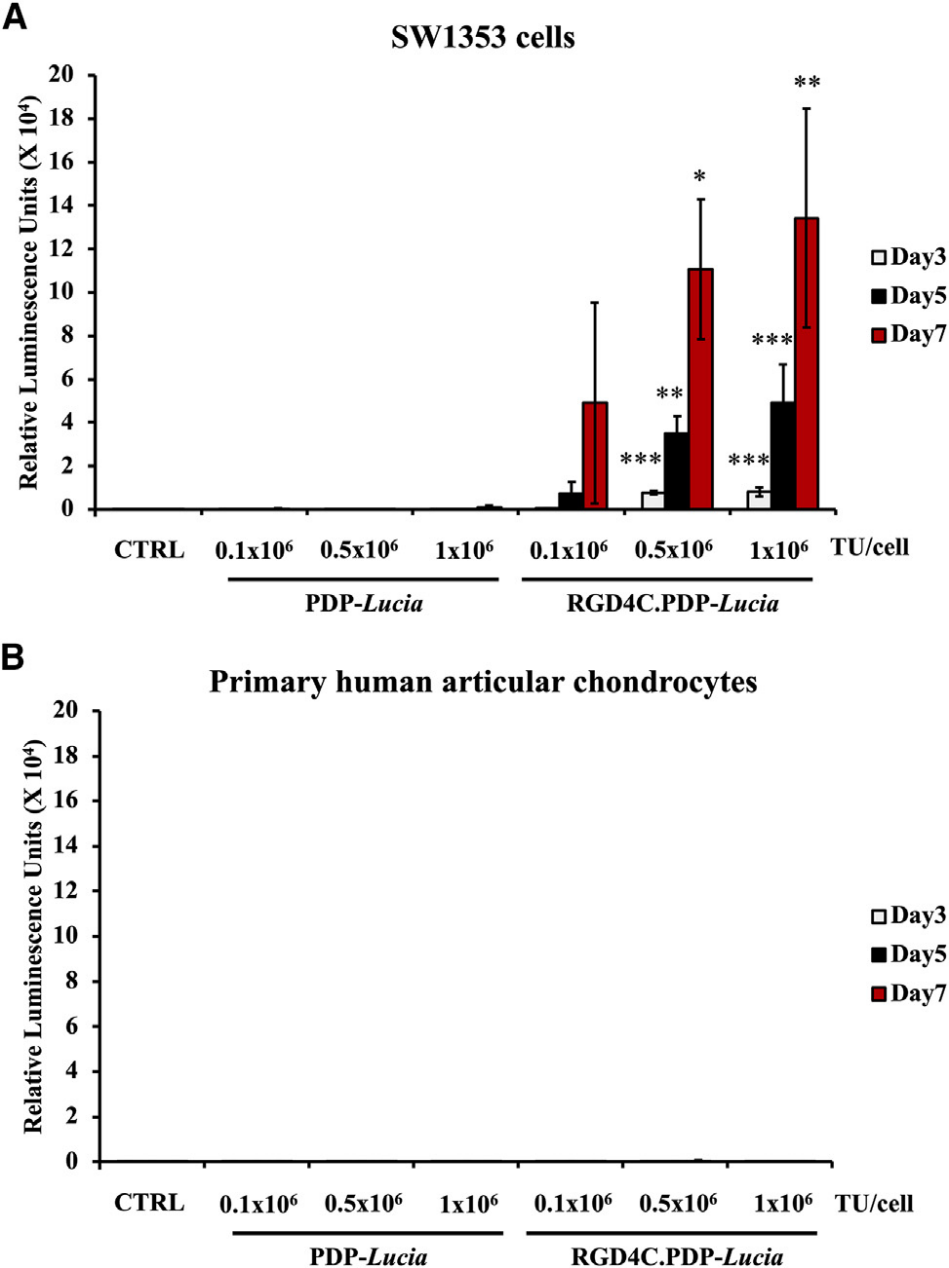
Targeted gene delivery by RGD4C.PDP (A) Human CS SW1353 cells were treated with either the tumor-targeted RGD4C.PDP-*Lucia* or non-targeted PDP-*Lucia* control with increasing doses of the particles (0.1 × 10^6^, 0.5 × 10^6^, and 1 × 10^6^ TU/cell). Untreated cells were also included as control (CTRL). Lucia expression was measured from the cultured media of transduced cells on days 3, 5, and 7 after vector transduction. (B) HACs were also used for PDP transduction. Results are shown as mean ± SEM of triplicate wells of one representative experiment. All experiments were repeated three times. ∗*p* < 0.05, ∗∗*p* < 0.01, ∗∗∗*p* < 0.001.

To investigate transduction efficiency of the RGD4C.PDP in CS cells, we engineered a vector carrying a transgene encoding a secreted luciferase reporter gene, *Lucia*.^15^ SW1353 and HAC cells were treated with two different PDP particles, which either targeted RGD4C.PDP-*Lucia* or non-targeted PDP-*Lucia*, lacking RGD4C and used as a control. The PDP particles were applied at increasing doses of 0.1 × 10^6^, 0.5 × 10^6^, and 1 × 10^6^ transduction units (TU)/cell and transgene expression was evaluated over a time course at days 3, 5, and 7 after transduction, by measuring the luciferase activity in the culture media. Expression levels of Lucia in SW1353 cells treated with the tumor-targeted RGD4C.PDP-*Lucia* significantly increased over time in a dose-dependent manner, as shown in Figure 3A. In contrast, no expression of Lucia was detected in SW1353 cells incubated with the non-targeted PDP-*Lucia* vector. Moreover, importantly, no Lucia activity was observed in normal HACs treated with both targeted RGD4C.PDP-*Lucia* or non-targeted PDP-*Lucia* (Figure 3B). These data show that the RGD4C.PDP selectively targets gene delivery to SW1353 CS cells, mediated by the tumor targeting RGD4C ligand, while sparing the normal chondrocytes.

### TRAIL receptor-2, DR5, is highly expressed on CS cells, but not on normal HACs

TRAIL initiates apoptosis through the engagement of its death receptors.^19^ There are four types of receptors, including TRAIL receptors 1, 2, 3, and 4. TRAIL promotes apoptosis by binding with TRAIL receptors 1 or 2 also known as death cell receptors 4 and 5 (DR4 and DR5), respectively. In contrast, TRAIL receptors 3 and 4 are decoy receptors that impede the death cell cycle.^19^ Targeting tumor cell death by TRAIL requires DR4 or DR5 expression on their cell surface for efficient *TRAIL* gene therapy.

**Figure 4:**
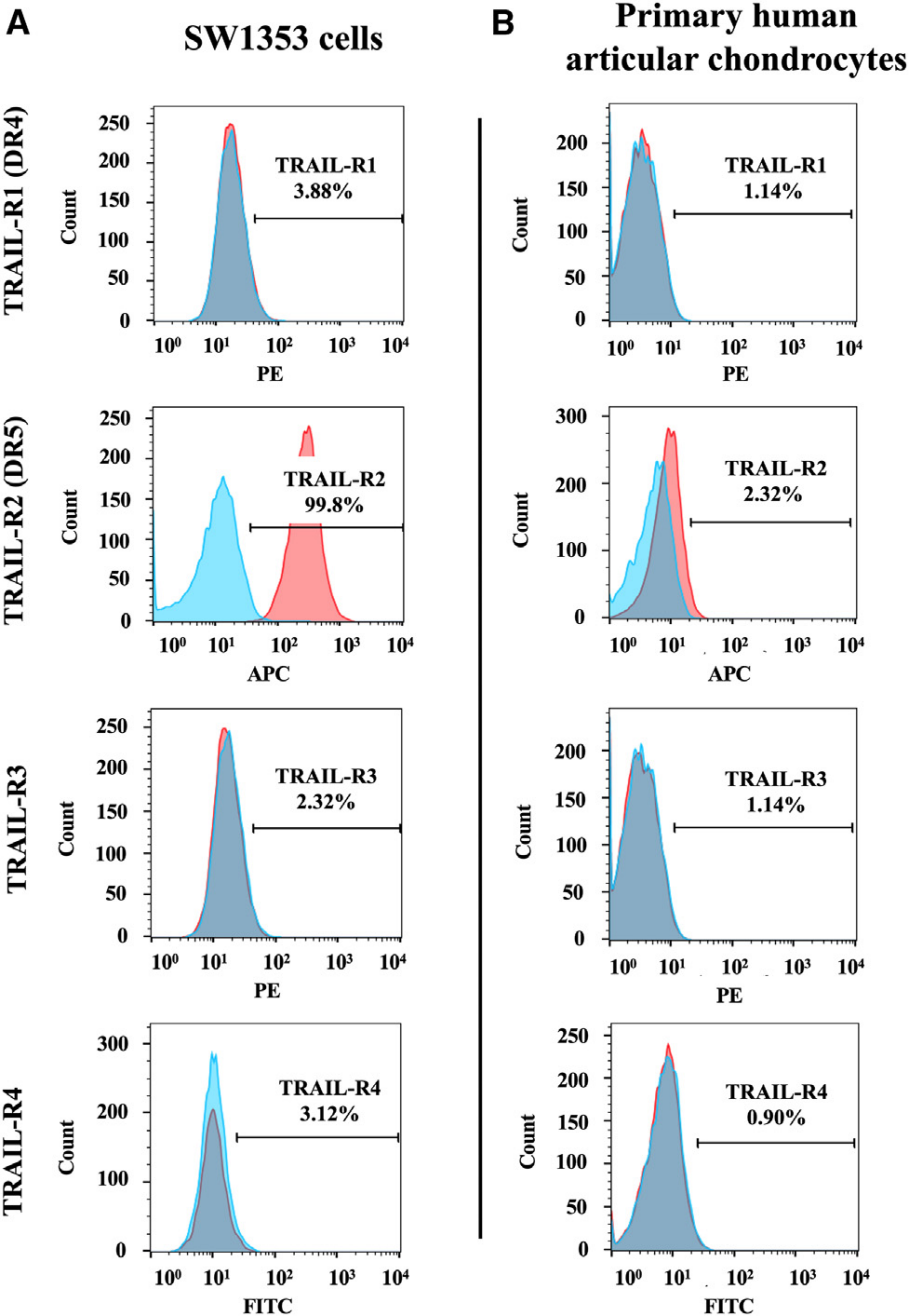
Evaluation of expression of TRAIL receptors in human CS cells and normal articular chondrocytes Flow cytometry analysis was performed on (A) human SW1353 CS cells and (B) HACs, which were stained for TRAIL-R1 (DR4), TRAIL-R2 (DR5), TRAIL-R3, and TRAIL-R4 (diluted 1:100 in FACS buffer). The expression was measured by flow cytometry and analyzed by FlowJo software. Unstained cells were used as control. The FACS results show the difference between stained and control groups.

We sought to investigate the expression of these receptors by staining SW1353 and primary human chondrocytes with specific antibodies to each TRAIL receptor. Data from flow cytometric analysis showed that 100% of SW1353 cells express DR5, even though there is no expression of DR4 (Figure 4A). Remarkably, neither decoy receptor, TRAIL-R3 and TRAIL-R4, were detected in SW1353 tumor cells (Figure 4A). Additionally, these receptors were barely detectable in normal HACs, with only 2% of chondrocytes expressing the DR5 death receptor (Figure 4B). Altogether, CS SW1353 is a suitable cell model for *TRAIL* cytokine gene therapy mediated by RGD4C.PDP-*sTRAIL*.

### Expression of TRAIL induces death of human CS cells

**Figure 5:**
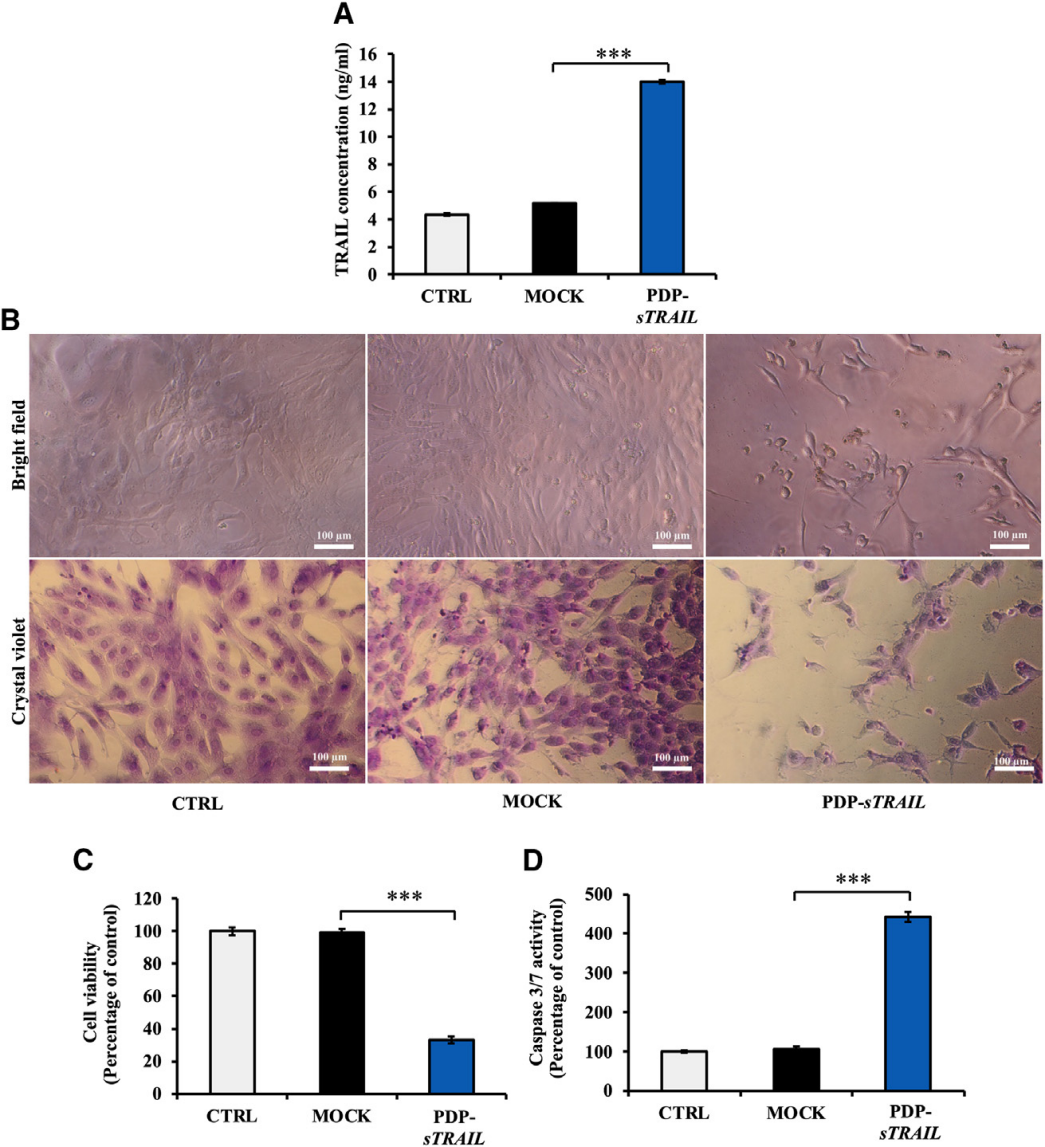
*In vitro* cell death of CS mediated by a PDP DNA construct carrying a recombinant sTRAIL DNA (PDP-*sTRAIL*) Human CS SW1353 cells at 60% confluency on 96-well plates were transfected with 6 μg DNA of PDP-*sTRAIL* plasmid using the FuGENE6 reagent. (A) Quantification of the secreted sTRAIL in the culture medium of cells by ELISA. (B) Brightfield imaging and crystal violet staining of cells showing CS cell morphology and confluence on day 2 after transfection. Scale bar, 100 μm. (C) Evaluation of cell death using a Cell Titer-Glo cell viability assay kit. (D) Analysis of caspase 3/7 activity using a Caspase-Glo 3/7 Assay System. Results are shown as mean ± SEM of triplicate wells of one representative experiment and compared with the MOCK transfection control group. All experiments were repeated twice. ∗∗∗*p* < 0.001.

TRAIL is expressed as a transmembrane protein that is then cleaved from the cell surface into a soluble homotrimer that binds to death receptors to induce cancer cell death.^24^ We have redesigned TRAIL to bypass this process and to express a direct secreted sTRAIL by generating a recombinant human *TRAIL* DNA sequence in which the signal peptide of TRAIL was removed and replaced by the leading peptide sequence of interleukin-2 (IL-2) to produce a secreted sTRAIL protein following PDP transduction. Next, to evaluate the secretion of the newly designed sTRAIL, we transfected SW1353 cells with PDP-*sTRAIL* DNA construct and quantified the sTRAIL protein level in the culture media of transfected cells by ELISA. As shown in Figure 5A, sTRAIL was highly detected in the culture media of cells transfected with the PDP-*sTRAIL* at approximately 14 ng/mL and was more than 3-fold higher than the sTRAIL levels of the control non-transfected and mock-transfected cells.

Importantly, the production of TRAIL induced the death of cancer cells and a dramatic change in cell morphology from fibroblast-like cells into round-shaped cells that detached from the adherent surface (Figure 5B), whereas non-transfected and mock-transfected cells remained healthy and unchanged morphologically (Figure 5B). Next, the cell viability assay revealed approximately 70% of cell death in the PDP-*sTRAIL* transfection group when compared with control non-transfected and mock-transfected groups (Figure 5C). These results demonstrate the efficacy of the newly designed sTRAIL to induce CS cell death.

TRAIL activates apoptotic cell death through the caspase signaling cascade. It binds to DR4 and DR5 receptors and triggers apoptosis via caspase 8 and caspase 3/7, respectively. Thus, since approximately 100% of SW1353 CS cells express DR5 (Figure 4A), we evaluated caspase 3/7 and detected a significant increase of its activity by more than 4-fold when compared with controls, non-transfected cells and mock transfection (Figure 5D). These data demonstrate that the PDP-*sTRAIL* transfection of CS cells produces active secreted sTRAIL cytokine that subsequently induces tumor cell death via the caspase pathway.

### RGD4C.PDP-*sTRAIL* particle carrying the *sTRAIL* DNA mediates the death of CS cells, but not human primary chondrocytes

**Figure 6:**
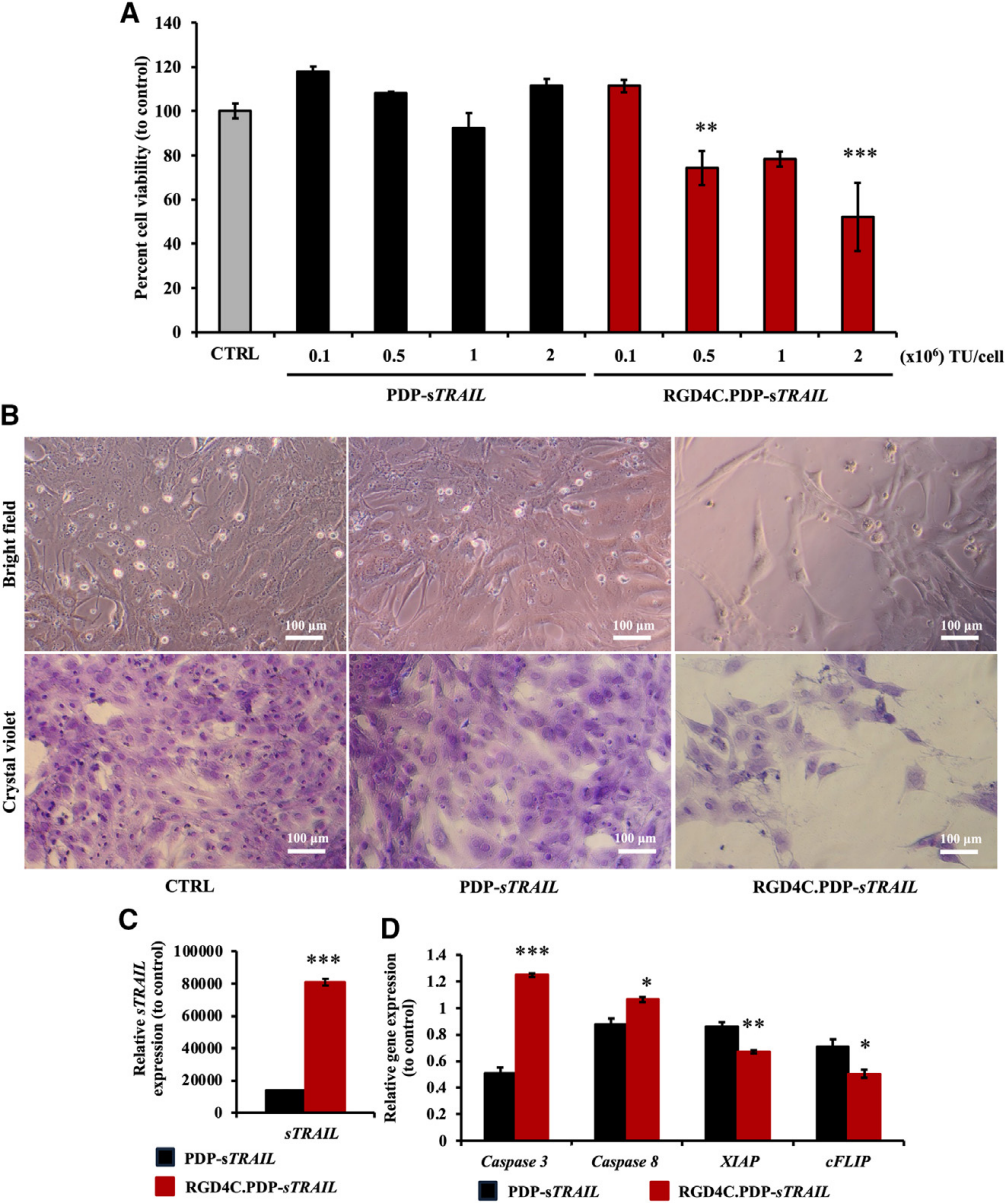
*In vitro* treatment of human CS cells with the RGD4C.PDP-*sTRAIL* particles (A) SW1353 cells were transduced with either targeted RGD4C.PDP-*sTRAIL* or PDP-*sTRAIL* at 0.1, 0.5, 1, and 2 × 10^6^ TU/cell. At day 7 after transduction, cells were analyzed for cell viability using CellTiter-Glo Luminescent Cell Viability Assay. (B) microscopic imaging of cells upon staining with crystal violet. Images were taken using 10× objective lens. Scale bar, 100 μm. (C) Evaluation of *sTRAIL* gene expression by RT-qPCR, at day 3 after transduction with either targeted RGD4C.PDP-*sTRAIL* or non-targeted PDP-*sTRAIL* at 0.5 × 10^6^ TU/cell. (D) Analysis of expression of apoptotic caspases 3 and 8 as well as anti-apoptotic *XIAP* and *cFLIP* genes. All results are shown as mean ± SEM of triplicate wells of one representative experiment and compared with PDP-*sTRAIL* treated group. ∗*p* < 0.05, ∗∗*p* < 0.01, ∗∗∗*p* < 0.001.

Next, we produced RGD4C.PDP-*sTRAIL* particles using the PDP-*sTRAIL* DNA construct. The RGD4C.PDP-*sTRAIL* particles were designed to display five copies of the RGD4C peptide motif on its pIII minor coat proteins, and EEP H5WYG on the recombinant pVIII major coat proteins to facilitate endosomal escape of PDP particles and enhance gene delivery. PDP particles without RGD4C, PDP-*sTRAIL*, were also produced as non-targeted controls. We transduced SW1353 cells with increasing vector doses of 0.1 × 10^6^, 0.5 × 10^6^, 1 × 10^6^, and 2 × 10^6^ TU/cell and analyzed cell death at day 7 after transduction. Significant cell killing was observed in the RGD4C.PDP-*sTRAIL* treatment group as compared with the absence of cell death in untreated cells or cells treated with the control non-targeted particles (Figures 6A and 6B). Next, we extracted mRNA from the transduced cells and performed RT-qPCR to quantify expression of *sTRAIL* at the transcription level. Remarkably, as shown in Figure 6C, transcription of *TRAIL* in the RGD4C.PDP-*sTRAIL* treatment group was approximately 80,000-fold higher than in control cells treated with the non-targeted PDP-*sTRAIL* particles. We also evaluated caspases 3 and 8 and detected increased gene expression of both caspases upon treatment with the RGD4C.PDP-*sTRAIL* (Figure 6D). After that, we investigated expression of anti-apoptotic genes, including an X-linked inhibitor of apoptosis protein; *XIAP* and cellular FLICE (FADD-like IL-1β-converting enzyme)-inhibitory protein; *cFLIP* and found a significant decrease in their expression in the RGD4C.PDP-*sTRAIL* treatment group (Figure 6D).

Next, we tested the effect of RGD4C.PDP-*sTRAIL* on normal human chondrocytes following treatment with RGD4C.PDP-*sTRAIL* or non-targeted PDP-*sTRAIL* at 0.1 × 10^6^ and 0.5 × 10^6^ TU/cell. No morphological changes (Figure S3A) or cell death (Figure S3B) were observed in these normal chondrocytes. These findings show that the RGD4C.PDP-*sTRAIL* vector generates significant production of sTRAIL in CS cells, subsequently leading to cancer cell death without harming the normal chondrocytes.

### RGD4C.PDP-*sTRAIL* treatment of human CS established in mice

**Figure 7:**
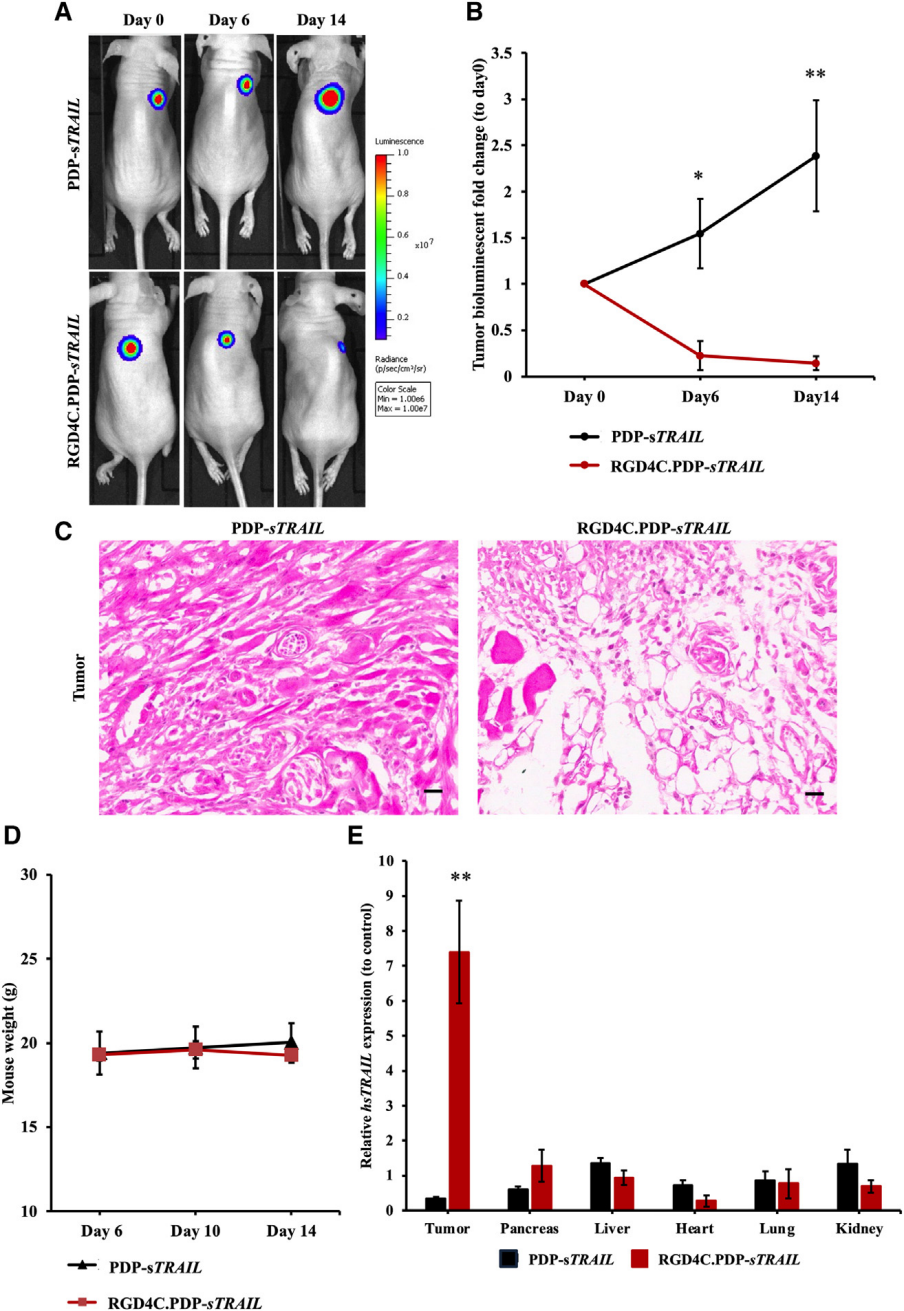
*In vivo* treatment of CS SW1353-bearing mice BALB/c *nu/nu* mice with established subcutaneous xenografts were intravenously administrated with either targeted RGD4C.PDP-*sTRAIL* or non-targeted PDP-*sTRAIL* at a dose of 5 × 10^10^ TU per mouse, twice a week for 14 days. (A) Representative tumor-bearing mice, from all experimental groups, imaged using the In Vivo BLI System at day 0 (before treatment initiation) and day 6 and day 14 after vector administration. (B) Tumor luminescence values shown as fold change between pre-treatment day 0 and post-vector treatment day 14. (C) Microscopic images of the H&E-stained tumors upon treatment with targeted RGD4C.PDP-*sTRAIL* or non-targeted PDP-*sTRAIL*. Scale bar, 20 μm. (D) Average weights of SW1353 tumor-bearing mice, from all experimental groups, measured on days 6, 10, and 14 after vector injection. (E) Evaluation of *sTRAIL* gene expression in tumors versus normal organs, from tumor-bearing mice, after intravenous administration of either targeted RGD4C.PDP-*sTRAIL* or non-targeted PDP-*sTRAIL*. Results are shown as mean ± SEM. ∗*p* < 0.05, ∗∗*p* < 0.01.

To establish tumors, immunodeficient BALB/c *nu/nu* mice were subcutaneously implanted with human CS SW1353 cells stably expressing the firefly luciferase, *Luc,* reporter imaging gene. *Luc* expression in tumors, luminescence, was monitored and evaluated overtime by repetitive bioluminescent imaging (BLI) of living tumor-bearing mice to detect tumors and to monitor tumor growth, viability, and tumor response to treatment as we previously reported.^15^^,^^16^ Next, tumor-bearing mice were intravenously injected with either targeted RGD4C.PDP-*sTRAIL* or non-targeted PDP-*sTRAIL* at a dose of 5 × 10^10^ TU per mouse, twice a week. Next, we carried out serial BLI of Luc in whole tumor-bearing mice in each group. Mice administered RGD4C.PDP-*sTRAIL* showed clear decrease of tumor size from day 0 to day 14 after treatment (Figure 7A), followed by a significant decrease in tumor luminescence over time, reflecting tumor viability, in mice treated with the RGD4C.PDP-*sTRAIL* (Figure 7B). In contrast, tumors receiving non-targeted PDP-*sTRAIL* grew larger in size and showed increased viability overtime, approximately 2.5-fold, from day 0 to day 14 (Figures 7A and 7B). These finding were confirmed by hematoxylin and eosin (H&E) staining of the tumor sections after therapy, revealing a clear damage to tumors following systemic gene therapy with RGD4C.PDP-*sTRAIL* compared with non-targeted PDP-*sTRAIL* (Figure 7C). The mice were also weighed twice a week during treatment and no difference in weight was detected between all experimental groups, subsequently showing no effect of the treatment on animal weights (Figure 7D). These data demonstrate that the RGD4C.PDP-*sTRAIL* can be used for targeted systemic *TRAIL* cytokine therapy against human CS.

### Biodistribution of *sTRAIL* expression in tumor-bearing mice following intravenous treatment with RGD4C.PDP*-sTRAIL*

We investigated the biodistribution of *sTRAIL* expression in mice with established CS to analyze expression of *sTRAIL* in tumors versus vital normal organs following treatment with the RGD4C.PDP*-sTRAIL*. Thus, immunodeficient mice with established human CS derived from SW1353 cells received intravenous injections of targeted RGD4C.PDP*-sTRAIL* or non-targeted PDP*-sTRAIL* at 5 × 10^10^ TU/mouse, twice a week. Tumor and normal organs, including pancreas, liver, heart, lungs, and kidneys, were collected and processed, then qPCR was performed to quantify the expression of *sTRAIL* mRNA in the collected tissues (Figure 7E). Remarkably, treatment of tumor-bearing mice with the targeted RGD4C.PDP*-sTRAIL* resulted in significant expression of *sTRAIL* in CS, with approximately a 7.4-fold increase when compared with the non-targeted PDP*-sTRAIL* group, which showed no detectable expression of *sTRAIL* in tumors (Figure 7E).

Moreover, the expression of *sTRAIL* in normal organs by RGD4C.PDP*-sTRAIL* was either similar or lower than that of the non-targeted PDP*-sTRAIL* group. Next, H&E staining of these normal organs did not reveal any tissue damage or abnormalities following repeated PDP treatment (Figure S4). Altogether, these findings confirm the tumor selectivity of systemic RGD4C.PDP*-sTRAIL* treatment and its safety in mice with established human CS.

## Discussion

In this study we introduced a novel approach to CS treatment by targeted TRAIL gene therapy as an alternative to the current limited CS therapy. To target the expression of TRAIL in CS, we used a PDP^15^ as a gene delivery vector to deliver a DNA sequence encoding a secreted version of TRAIL. The PDP delivery system has some potential advantages over existing viral vectors. The PDP particles can be easily produced using an *E. coli* bacterial host; production is rapid and cost effective, and scale-up is feasible. Although phage has no native tropism for mammalian cells and tissues, we can use phage display to generate hybrid coat proteins and direct the phage toward specific receptors in cancer, resulting in a tumor-targeted PDP particle displaying the RGD4C ligand that binds the αvβ3 integrin. This cell surface receptor is crucial for angiogenesis and tumor metastasis and is upregulated in newly synthesized blood vessels and tumor cells in most types of cancer.^25^ There have been reports on αvβ3 expression in CS and its association with tumor cell migration,^26^^,^^27^ as well as CS metastasis to the lungs.^28^ The other αvβ5 integrin receptor of RGD4C has also been associated with CS migration.^17^ Therefore, we reasoned that RGD4C.PDP expressing the RGD4C ligand as a fusion with the phage pIII minor coat proteins is a potential candidate for targeting CS. Next, to enhance gene delivery, the RGD4C.PDP particle was further modified to display the H5WYG, a histidylated fusogenic peptide with endosomal buffering capacity, on the recombinant pVIII major coat proteins to boost PDP escape from the endo-lysosomal pathway, as we have reported.^18^

Herein we showed that human CS cells express the αvβ3 and αvβ5 integrin receptors of the RGD4C.PDP. Then we used a vector encoding a secreted reporter luciferase (*Lucia*) as a simple way to quantify and demonstrate gene expression from RGD4C.PDP, and its selectivity, by measuring the Lucia activity in the cell culture medium without the need to lyse the cells, unlike the conventional *luciferase* reporter gene.^15^ No integrin receptor and no gene delivery were detected in normal primary chondrocytes, confirming our previous findings on the tumor selectivity of RGD4C.PDP.^15^ We also found that the treatment of human CS cells *in vitro* with the RGD4C.PDP-*sTRAIL* expressing a secreted version of TRAIL resulted in significant production and secretion of TRAIL in the culture media of cells and caused cancer cell death without any effect on normal primary chondrocytes. CS cell death by RGD4C.PDP-*sTRAIL* was associated with increased expression of *caspases 3* and *8* and decreased of expression of anti-apoptotic *XIAP* and *cFLIP* genes, confirming the TRAIL-mediated cancer cell death by apoptosis.^19^

*In vivo* repeated intravenous administrations of RGD4C.PDP-*sTRAIL* in a mouse model of human CS resulted in the eradication of tumor size and tumor viability. We also confirmed selective expression of *sTRAIL* in CS in mice, while avoiding the normal organs, following systemic treatment with the targeted RGD4C.PDP-*sTRAIL*. The safety, biodistribution, and tumor selectivity of gene delivery by RGD4C-derived phage vectors was comprehensively reported in our previous studies.^11^^,^^12^^,^^13^^,^^14^^,^^15^^,^^16^^,^^18^^,^^22^ Furthermore, it is noteworthy to mention that the M13 phage (parent of the PDP) carrying a peptide library on the capsid was injected intravenously and repeatedly into cancer patients without causing any unfavorable clinical side effects.^29^

TRAIL is well known as a significant activator of the cell death pathway. TRAIL can initiate apoptosis through engagement of its death receptors, which are highly expressed in many types of tumor cell lines, but absent in most normal cells. TRAIL death receptors are highly expressed in bone sarcomas and can thus be used to trigger apoptosis in tumor cells selectively.^30^ In a phase 1 clinical trial, the anticancer efficacy of recombinant human TRAIL (rhTRAIL [dulanermin]) was evaluated in patients with advanced cancer. Overall, rhTRAIL caused a partial responses in two of five CS patients.^31^ Remarkably, both CS patients responded to rhTRAIL monotherapy; in one of them, long-term survival was completed with a combination of prolonged rhTRAIL treatment and surgery.^32^ Overall, although clinical trials in various cancer types reported a decent safety profile of TRAIL, its effectiveness was limited. Unfortunately, rhTRAIL has an extremely short half-life, namely, 3–5 min in mice and 30–60 min in primates, impeding further development.^33^ Brief, the short half-life, poor tumor delivery efficacy, and resistance to TRAIL monotherapy are the three main limitations of TRAIL-based treatment.^34^

In this context, following homing and entry into cells, the RGD4C.PDP-*sTRAIL* should allow continuous gene expression and secretion of TRAIL into the tumor microenvironment. Ultimately, this should boost TRAIL release in the target tumor tissue and its bioavailability to its death receptors, and subsequently overcome cancer resistance to TRAIL. Moreover, following tumor transduction by RGD4C.PDP-*sTRAIL*, secreted TRAIL from transduced cells can bind to its death receptors on surrounding non-transduced cancer cells, causing their death and inducing a bystander effect as reported.^35^

In conclusion, our preclinical investigation in human CS yields important findings on the potential of RGD4C.PDP to overcome the limitations of TRAIL in cancer therapy paving the way toward targeted systemic therapy with TRAIL in CS patients.

## Materials and methods

### Construction and production of PDP

A PDP was constructed by engineering the M13 phage genome to display the double cyclic RGD4C ligand (ACDCRGDCFCG), as the tumor homing peptide, on the pIII minor coat proteins. In addition, PDP was modified to display the endosome escape peptide H5WYG on the recombinant pVIII coat proteins. PDP also carries a mammalian transgene cassette, flanked by AAV2 *ITRs* cis elements, and under the control of the cytomegalovirus, *CMV* promoter (Figure 1A). The *sTRAIL* sequence flanked by EcoRI and BamHI restriction sites was first digested with EcoRI and BamHI restriction enzymes, respectively, then ligated into the PDP, which was also digested with EcoRI and BamHI. RGD4C.PDP-*sTRAIL* was produced in an *E*. *coli* TG1 host, then purified as we reported.^15^ The PDP was sterile-filtered through 0.45-μm PVDF filter, then quantified as functional bacterial TUs by colony counting of host bacteria.

### Cell culture

Human CS cell line (SW1353) was purchased from the American Type Culture Collection. Primary HACs were separated from normal cartilage of non-osteoarthritic joints at Maharaj Nakorn Chiang Mai Hospital with informed consent and local ethical committee approval (Ethic approval no. ORT-11-09-16A-14). SW1353 and HAC cells were cultured in DMEM (Sigma) supplemented with 10% fetal bovine serum (FBS) (Sigma), penicillin (100 U/mL) (Sigma), streptomycin (100 μg/mL) (Sigma), and L-glutamine (2 mM) (Sigma), and maintained in a humidified atmosphere of 37°C in a 5% CO_2_. HAC cells were used for up to four passages.

### Flow cytometry

SW1353 and HAC cells were detached using a cell dissociation buffer (Life Tech) and washed twice with PBS. The cells’ number was then adjusted to 1 × 10^6^ cells/mL in cold fluorescence-activated cell sorting (FACS) buffer.

For the expression of integrins, cells were stained with the heterodimer αvβ3 or αvβ5 antibodies (diluted 1:50 in FACS buffer, R&D systems) for 30 min at room temperature, then washed three times with the FACS buffer. After that, cells were stained with Alexa Fluor 488 conjugated mouse IgG secondary antibody diluted 1:1,000 in 2% BSA in PBS (Thermo Fisher Scientific) for 30 min at room temperature, then washed three times with FACS buffer. The expression was analyzed by Flow cytometry and FlowJo software (BD Biosciences). Cells stained with the secondary antibody only were used as a control.

For the expression of the death receptors, the cells were stained with fluorescent dyes-conjugated anti-human CD261 (TRAIL-R1, dilution 1:100, BioLegend), CD262 (TRAIL-R2, dilution 1:100, BioLegend), CD263 (TRAIL-R3, dilution 1:100, Miltenyi Biotec), or CD264 (TRAIL-R4, dilution 1:100, Molecular Probes) for 30 min at room temperature in the dark, then washed three times with 1% BSA in PBS. The expression was measured by flow cytometry, and analysis conducted by FlowJo software (BD Biosciences). Unstained cells were used as controls.

### Immunofluorescent staining of integrins in cells

Human CS SW1353 cells and primary articular chondrocytes were stained for αv, β3 and β5 integrins on poly-D-lysine coated coverslips as previously reported.^12^ Cells were viewed and photographed using a Nikon Eclipse TE2000-S fluorescence microscope.

### *In vitro* cell transfection

Human CS cells (SW1353) were seeded into six-well plates and grown for 24 h to reach 60%–70% confluence. The cells were transfected with 6 μg DNA of phage vector encoding a secreted form of TRAIL, by using the FuGENE6 reagent (Promega) for 24 h at 37°C, then the medium was replaced with 1% FBS DMEM and the cells were incubated at 37°C to allow transgene expression. At day 2 after transfection, the amount of secreted TRAIL in the culture medium was measured by ELISA (BioLegend). Cell death was microscopically observed, then cell viability was determined by CellTiter-Glo cell viability assay kit (Promega). Caspases 3/7 activities were evaluated by using the Caspase-Glo 3/7 Assay System (Promega) by following the manufacturer’s protocol and quantified using a GloMax Navigator Microplate Luminometer (Promega).

### *In vitro* cell transduction by PDP

SW1353 and HAC cells were plated into well-plates and cultured for 24 h to reach 60%–70% confluence. Before transduction, cell culture medium was replaced with serum-free medium for 2 h. The cells were transduced with PDP, diluted in serum free medium and incubated for 24 h at 37°C. Next, the medium was replaced with fresh culture medium, and cells or culture media were collected and subsequently analyzed.

### RNA purification and RT-qPCR

Cells were harvested and RNA extracted using an RNA extraction kit (PureLink RNA Mini Kit, Thermo Fisher Scientific) following the manufacturer’s protocol. One microgram of total RNA was converted to cDNA using a High Capacity cDNA Reverse Transcription Kit (Applied Biosystems, Thermo Fisher Scientific). The expression of *TRAIL* and apoptotic relative genes was quantified by qPCR using Powerup SYBR Green Master Mix (Thermo Fisher Scientific). All primers were obtained from Life Technologies. The lists of primer sequences are shown in Table S1. The level of gene expression was relative to that of control group using the 2^−ΔΔCT^ calculated method. *GADPH* (the house-keeping gene) was utilized as an internal control.

### Cell viability assays

#### CellTiter-Glo luminescent cell viability assay

The cells were incubated with CellTiter-Glo Reagent (Promega) with the same volume as the cell culture media, then mixed for 2 min on a shaker. The cells were then placed for 10 min at room temperature. Next, the mixture was moved to opaque-walled multi-well plates, then the signal was measured using GloMax Navigator Microplate Luminometer (Promega).

#### Crystal violet staining

The cells were rinsed twice in gentle running tap water, then air-dried. Subsequently, the cells were incubated with 0.5% crystal violet staining solution (Sigma) for 20 min on a shaker. Next, the cells were rinsed four times in gentle running tap water and air dried. The cells were finally added with distilled water and observed under a microscope.

### ELISA for TRAIL quantification

The ELISA plate was coated with standards (50 ng/mL to 0.39 ng/mL) or samples at room temperature for 24 h. The plate was washed four times with PBS 0.05% Tween, then added a casein-blocking buffer at room temperature for 2 h. Next, the plate was washed four times with PBS 0.05% Tween. After this step, the plate was added 2.5 μg/mL primary antibody diluted with the blocking agent and incubated at 4^ο^C overnight. The plate was washed four times with PBS 0.05% Tween, then added 1:2,000 secondary antibody diluted with the blocking agent and incubated at room temperature for 2 h. The plate was washed four times with PBS 0.05% Tween, then added with a highly sensitive TMB and left for color development. Finally, the reaction was stopped by adding 2 N H_2_SO_4_ then the absorbances were measured at 450 nm.

### Lentivirus production and generation of the SW1353-*GFP*-*Luc* cell line

The 293T17 cells were cultured on T-175 flasks in complete medium (10% FBS/DMEM supplemented with 1% L-glutamine, 1% Na pyruvate, and 1% non-essential amino acids [NEAA]) for 24 h. The medium was replaced with fresh culture medium 2 h prior to transfection. The plasmid DNA for lentivirus production was combined: pLIV-*GFP*-*luc*, packaging vector: psPAX2, and pMD2.G in deionized water. The mixture was gently added with CaCl_2_ and bubbled through HEPES-buffered saline, then mixed and incubated at room temperature for 30 min. The solution was mixed again prior to adding dropwise to the 293T17 cells and incubated at 37°C, 3% CO_2_ for 4 h, then washed twice with PBS. Subsequently, the cells were added DMEM complete medium containing sodium butyrate 1 mM final concentration and incubated at 37^ο^C, 3% CO_2_ overnight. At 24 hours after transfection, the medium was collected, then replaced with DMEM complete medium containing sodium butyrate and incubated for 24 h. The medium was collected 48 h after transfection. The lentivirus containing media were combined and centrifuged at 1,500 rpm for 10 min. The supernatant was collected and filtered through a 0.45-μm PVDF filter and kept at 4°C.

The SW1353 cells were cultured on T-175 flasks in complete medium (10% FBS/DMEM supplemented with 1% L-glutamine, penicillin, and streptomycin) at 37°C, 5% CO_2_ for 24 h. The lentivirus-containing medium was added to the cells for 24 h. Next, the medium was replaced with DMEM complete medium daily until the expression of GFP and luciferase transgenes were observed. The GFP-positive cells were sorted using FACS. Finally, the SW1353-*GFP*-*Luc* cells were cultured and expanded, and the expression of GFP and luciferase monitored before implantation to the mice.

### *In vivo* experiments in tumor-bearing mice

All processes were reviewed and approved by the Animal Welfare and Ethical Review Body (AWERB committee) at Imperial College London before a final approval by the Home Office UK. All experiments were performed in accordance with the Institutional and Home Office Guidelines, and under a granted Home Office-issued project license. BALB/c *nu/nu* mice, 6-week-old were obtained from Charles River Laboratories. Labeled human CS cells (SW1353-*GFP*-*Luc*) were implanted subcutaneously in mice at 2 × 10^6^ cells per mouse. Tumor-bearing mice were intravenously administrated with targeted (RGD4C.PDP-*sTRAIL*) or non-targeted (PDP-*sTRAIL*) particles at a dose of 5 × 10^10^ TU per mouse, twice a week. We monitored the body weight and luciferase expression in each group of tumor-bearing mice. Tumor growth was observed twice a week by BLI of luciferase using the In Vivo Imaging System (IVIS 100; Caliper Life Sciences). Mice were anesthetized, then administered 100 mg/kg of D-luciferin (Gold Biotechnology). The mice were then imaged using the IVIS. A region of interest was determined manually over the tumors to estimate signal intensities described as total photon counts per second per square centimeter (p/sec/cm^2^/sr). All mice were euthanasia by terminal perfusion through the heart at the end of the study.

### *In vivo* biodistribution of PDP in CS SW1353-bearing mice

BALB/c *nu/nu* mice, 6 weeks-old were purchased from Charles River Laboratories. Human CS cells labeled with the *GFP* and *firefly* *luciferase* genes (SW1353-GFP-*Luc*) were implanted subcutaneously in mice at 2 × 10^6^ cells per mouse. Tumor-bearing mice were intravenously administrated with targeted (RGD4C.PDP-*sTRAIL*) or non-targeted (PDP-*sTRAIL*) particles at a dose of 5 × 10^10^ TU per mouse, twice a week. Mice were terminated by terminal perfusion through the heart at the end of the study. Tumor and normal organs were collected, processed, and stained with H&E. Fresh collected tissues were added with cold lysis buffer with 1% β-mercaptoethanol and homogenized using a homogenizer (Minilys, Bertin Technologies SAS). RNA was extracted following the manufacturer’s protocol. qPCR was performed to detect the expression of *sTRAIL* in the tissues using Powerup SYBR Green Master Mix (Thermo Fisher Scientific). All primers were obtained from Life Technologies. The lists of primer sequences are shown in Table S1. The level of gene expression was relative to that of control group using the 2^−ΔΔCT^ calculated method. Human *ACTB* and mouse *ACTB* (the housekeeping genes) were utilized as internal controls.

### Negative staining for transmission electron microscopy of PDP

The formvar carbon coated 200 mesh grids were glowed discharged at 10 mA for 2 min. PDP particles were added on the grids, then left for 1 min and expelled using blotting paper. The grids were rinsed with filtered deionized water and expelled using blotting paper, then floated on a drop of filtered deionized water for 3 min and expelled using a blotting paper. Next, the grids were floated on drop of 1% glutaraldehyde for 5 min and expelled using blotting paper. The grids were negatively stained by applying 1% uranyl acetate to the grids for 4 min, then expelled using blotting paper. Images were taken using a transmission electron microscope (JEOL JEM-2010) and analyzed by using ImageJ software.

### Statistical analysis

All data are shown as mean ± SEM. We utilized independent *t* tests, one-way ANOVA, and Tukey’s HSD post hoc test for statistical analyses. Statistical significance is represented as *p* values, where ∗*p* < 0.05, ∗∗*p* ≤ 0.01, and ∗∗∗*p* ≤ 0.001. All statistical analyses were created using SPSS software.

## Data and code availability

The data supporting the findings of this study are available in the materials and methods section of this article. Data sharing is not applicable to this research article as no datasets were generated.

## Supplemental information

Document S1. Figures S1–S4 and Table S1

Document S2. Article plus supplemental information
