# Supplementary material for: Targeted treatment of chondrosarcoma with a bacteriophage-based particle delivering a secreted tumor necrosis factor-related apoptosis-inducing ligand
Source: Mol Ther Oncol. 2024 Apr 24;32(2):200805. doi: 10.1016/j.omton.2024.200805 (PMC11090904; doi:10.1016/j.omton.2024.200805)
Supplement: Document S1. Figures S1–S4 and Table S1 [file mmc1.pdf]

OMTON, Volume *32*

Supplemental information

Targeted treatment of chondrosarcoma with a bacteriophage-based particle delivering a secreted tumor necrosis factor-related apoptosis-inducing ligand

Aitthiphon Chongchai, Kaoutar Bentayebi, Grace Chu, Wenqing Yan, Sajee Waramit, Thanyaluck Phitak, Prachya Kongtawelert, Peraphan Pothacharoen, Keittisak Suwan, and Amin Hajitou

Table S1. Primer sequences for RT-qPCR

Gene	Primers (5’→3’)
Human sTRAIL	Forward: *CCAGAGGAAGAAGCAACACA*
	Reverse: *GAATAGATGTAGTAAAACCCTTTTTCA*
Human Caspase 3	Forward: *TGGTTCATCCAGTCGCTTTG*
	Reverse: *CATTCTGTTGCCACCTTTCG*
Human Caspase 8	Forward: *GCCTCCCTCAAGTTCCT*
	Reverse: *CCTGGAGTCTCTGGAATAACA*
Human XIAP	Forward: *AACCTTGTGATCGTGCCT*
	Reverse: *ACCCTGGATACCATTTAGC*
Human cFLIP	Forward: *GCTCACCATCCCTGTACCTG*
	Reverse: *CAGGAGTGGGCGTTTTCT*
Human GAPDH	Forward: *CCCCTTCATTGACCTCAACTAC*
	Reverse: *GATGACAAGCTTCCCGTTCTC*
Human ACTB	Forward: *CACCATTGGCAATGAGCGGTTC*
	Reverse: *AGGTCTTTGCGGATGTCCACGT*
Mouse ACTB	Forward: *CATTGCTGACAGGATGCAGAAGG*
	Reverse: *TGCTGGAAGGTGGACAGTGAGG*

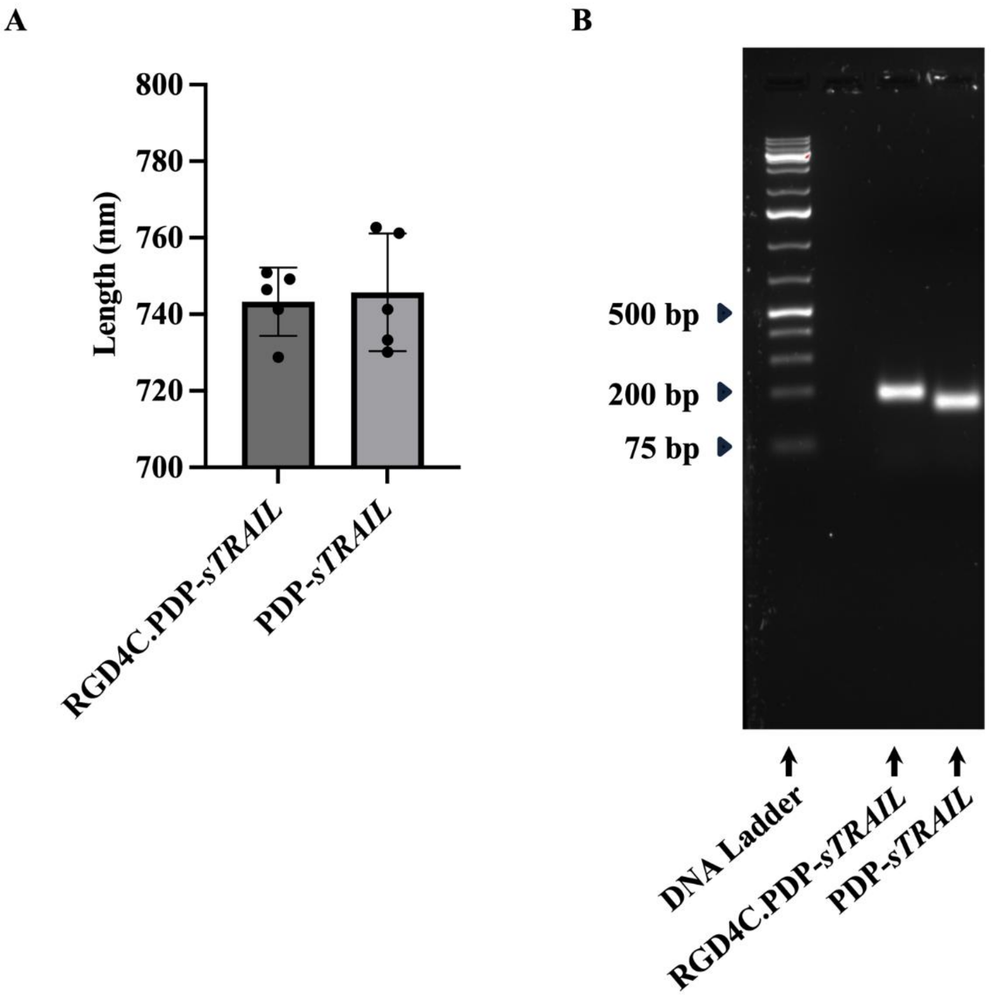

Figure S1. Physical characterisation of the vectors. **A**) Comparison of particle length of targeted RGD4C.PDP-*sTRAIL* and non-targeted PDP-*sTRAIL* using images from the transmission electron micrographs. The measurements were done using ImageJ software.

B) Agarose gel electrophoresis depicting the PCR products of the amplified pIII gene region containing the insertion site of the RGD4C ligand. The targeted RGD4C.PDP-*sTRAIL* carrying the RGD4C-encoding DNA sequence yields a larger PCR product (200 bps) than the non-targeted PDP-*sTRAIL* vector (173 bps).

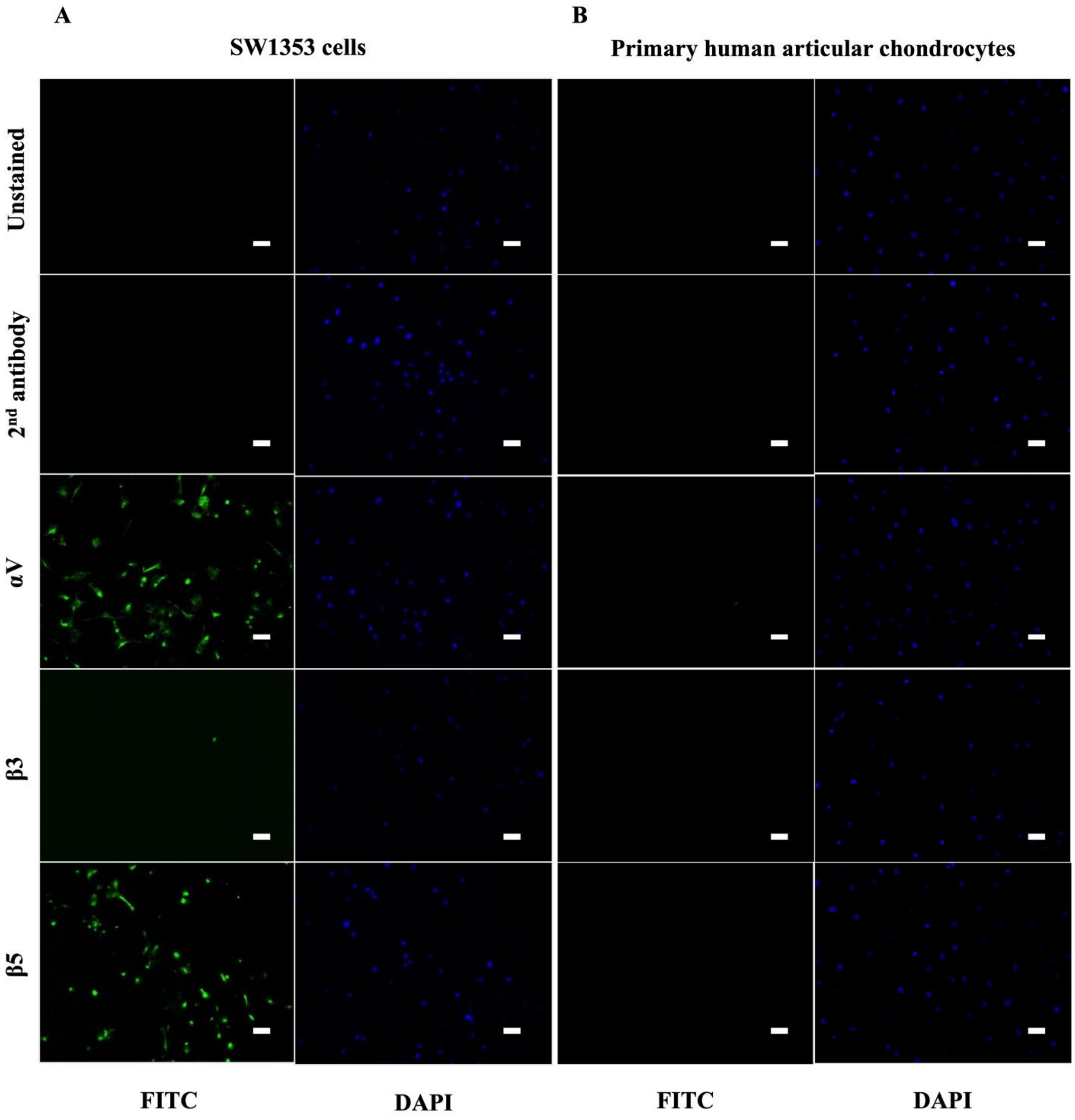

Figure S2. Immunofluorescent staining of αV, β3, and β5 integrins. **A**) Human chondrosarcoma SW1353 cells. **B**) Primary human articular chondrocytes. Cells were observed under a fluorescence microscope (10x) and analysed using ImageJ software. Cells treated with the secondary antibody alone or unstained cells served as controls. FITC (fluorescein isothiocyanate): integrin staining. Blue: DAPI (4′,6-diamidino-2-phenylindole), nuclear staining. Scale bar, 160 μm.

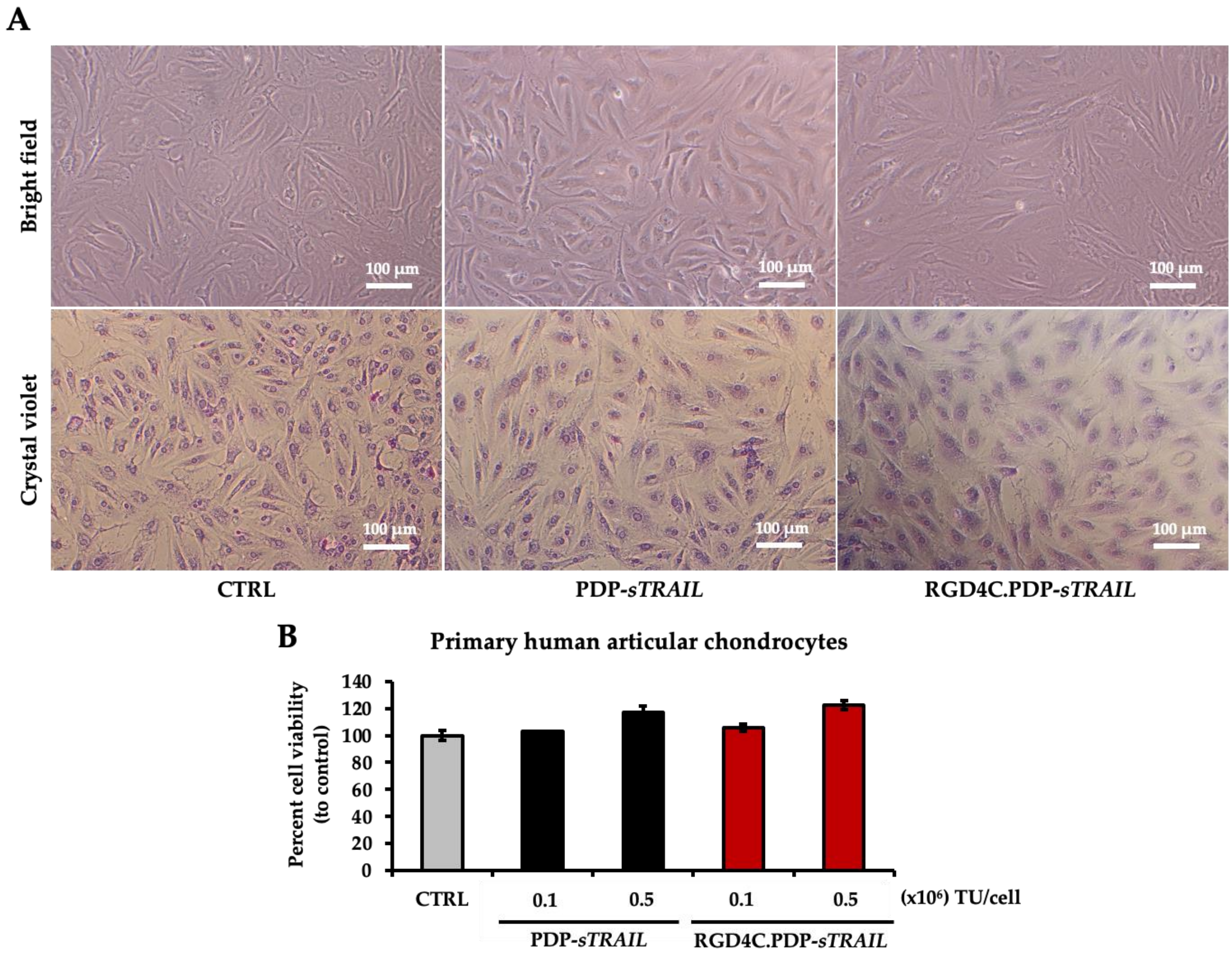

Figure S3. Evaluation of cell death in normal human articular chondrocytes upon exposure to targeted RGD4C.PDP-*sTRAIL* or non-targeted PDP-*sTRAIL*. (**A**) Phase contrast images showing normal morphology at day 7 post-transduction. (**B**) Evaluation of cell viability of the chondrocytes using CellTiter-Glo® Luminescent Cell Viability Assay. All results are shown as mean ± SEM of triplicate wells of one representative experiment and compared to PDP-*sTRAIL* treatment group.

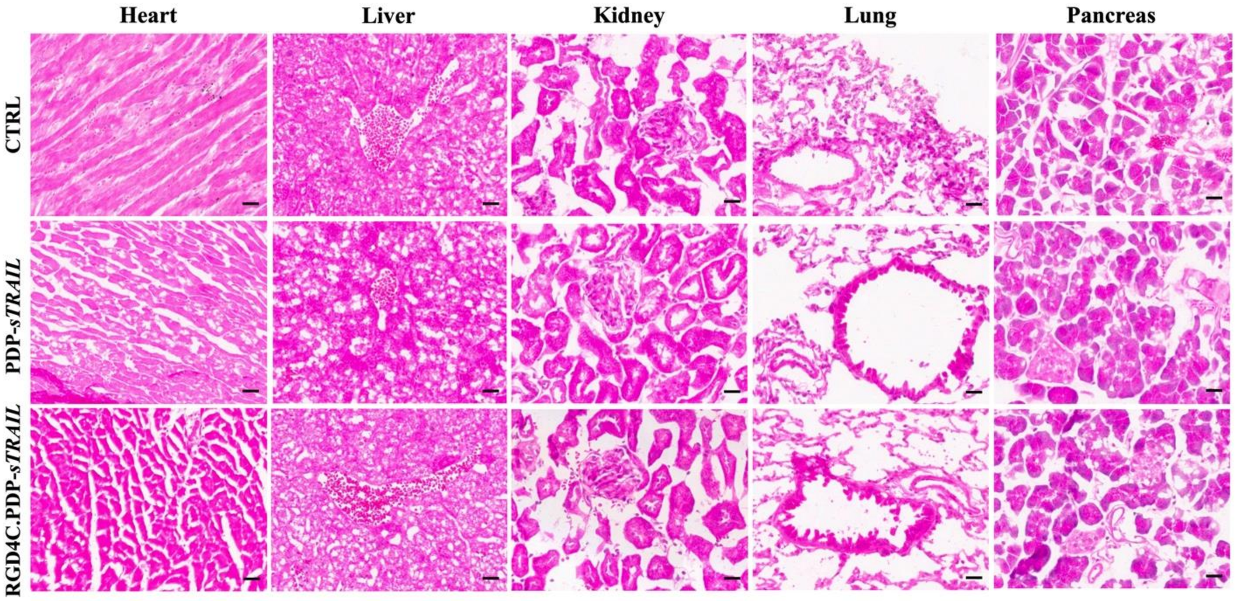

Figure S4. Microscopic imaging of H&E-stained sections of normal organs following treatment with targeted RGD4C.PDP-*sTRAIL* or non-targeted PDP-*sTRAIL*. Organs from control untreated mice (CTRL) were also stained with H&E. Scale bar, 20 μm.
